# Toward a Future Where Diabetes No Longer Limits Mesh Surgery

**DOI:** 10.1007/s00192-026-06675-8

**Published:** 2026-05-07

**Authors:** Rui Liang

**Affiliations:** Department of Anesthesiology, OB&GYN, College of Medicine, Ohio State University, 460 W. 12th Ave., Columbus, OH 43210 USA

**Keywords:** Pelvic reconstructive surgery, Mesh, Diabetes, Host response, Tissue quality

## Abstract

The use of synthetic mesh in urogynecologic pelvic reconstructive surgery has evolved substantially over the past three decades. Although advances in biomaterials, textile design, and surgical techniques have improved mesh outcomes, patient-specific factors remain insufficiently addressed. Among these, diabetes is common in women undergoing pelvic floor surgery and is associated with an increased risk of mesh-related complications. This review summarizes current perioperative glucose management in surgical populations and discusses its relevance to mesh implantation. Despite widespread reliance on HbA1c for preoperative assessment, reliable markers for predicting mesh outcomes are lacking. Intra- and postoperative glucose control also affects outcomes; however, optimal targets and their relationship to long-term implant success remain to be better defined. Patient management is further complicated by altered immune function driven by glycemic variability and hyperglycemic memory, alongside diabetes-related declines in tissue quality. This editorial advocates for a shift toward an individualized, precision medicine framework that integrates patient-specific factors and emphasizes the need for mechanistic studies, biomarker discovery, and computational modeling.

The use of synthetic mesh in urogynecologic pelvic reconstructive surgery has spanned nearly three decades, yet it remains a subject of debate. It has gone through a challenging path from adaption from hernia mesh to FDA regulation and to regained confidence toward a safer and more sophisticated applications in the field [1–5]. Since 2019, when the FDA ordered a halt to the sales of transvaginal POP meshes in the United States [5], newly reported complications have largely declined overall, although complications related to mid-urethral slings and legacy prolapse meshes continue to be observed [6, 7]. Thanks to mechanistic works on mesh materials, textile architecture, mechanical properties, microbiome interactions, and the refinement of surgical techniques, we have become better at designing and deploying meshes. Yet one major risk factor remains stubbornly beyond our direct control: the patient’s own biology.

The global rise of diabetes, driven largely by type 2 disease, is reshaping our medical practice, and pelvic floor surgery is no exception [8–10]. Because diabetes becomes more prevalent with age as well as pelvic floor dysfunctions in women, a substantial proportion of women we see in urogyne practice live with both conditions. Cohort studies suggest that roughly 10–15% of women undergoing mesh-augmented pelvic reconstructive surgery have diabetes as a comorbidity [11–19]. More than just a background diagnosis, diabetes acts as an independent risk for mesh complications [11–19]. In studies from the transvaginal prolapse mesh era, women with diabetes faced up to sevenfold higher risks of mesh exposure and erosion than women without [14]. For an individual surgeon sitting with a patient in the clinic, the question of whether and when to implant mesh in a woman with diabetes remains a nuanced, and at times difficult, decision.

Mesh implantation surgery appears to have low tolerance for chronically poor glucose control—but how good is “good enough”? HbA1c, the most widely used marker before surgery, offers only partial guidance [20–22]. Some clinicians favor stricter thresholds (< 7%) before mesh implantation, while others accept higher values, citing feasibility and the lack of conclusive data. Observational studies across surgical disciplines associate HbA1c levels ≥ 7–8% with higher rates of infection and wound complications [20–26]. Yet these studies largely address short-term outcomes and do not adequately capture the prolonged biological interactions between host tissue and a permanent implant. Continuous glucose monitoring is also gaining traction as an important adjunct, reflecting an increasing awareness of the negative impact of glucose fluctuation and variability [27]. Yet, its value in predicting long-term implant outcomes remains to be investigated. Adding to the uncertainties is an intriguing concept of hyperglycemic memory: even brief episodes of hyperglycemia can leave durable epigenetic and functional imprints on innate immune cells such as macrophages [28–30]. These cells are among the first responders that react to the surgical injury and mesh implantation. It is plausible that this “trained” innate immunity shapes long-term host–mesh interactions, a possibility that clearly invites further investigation. Taking together, more scientific research is needed to answer this seemingly simple question.

Inside the operating room, the glucose management is complicated by anesthesia, stress hyperglycemia, surgical approaches, and comorbidities. Professional associations and societies recommend relatively broad intraoperative glucose control targets from 80 to 180 mg/dL [31]. This corresponds to studies showing that glucose > 180 mg/dL is consistently associated with higher rates of major adverse events, while pushing glucose too tightly below ~ 100–110 mg/dL induces hypoglycemia without clear benefits [32–37]. There is no one-size-fits-all protocol—certainly not for mesh implantation. What we do know is that hyperglycemia is generally unfriendly and should be managed as best as we can [38, 39]. What we do not yet know is how intraoperative glycemic patterns impact short- and long-term mesh outcomes, calling for studies to fill the knowledge gap.

The postoperative course adds another layer. Across diverse surgical populations, postoperative glucose level ≥ 140 mg/dL within days to weeks are associated with increased complications, with risks rising further above ~ 180 mg/dL and, in some studies, ≥ 225 mg/dL for severe wound problems and mortality [22, 40–44]. For mesh implantation, these thresholds are a reasonable starting point. In a study we conducted to evaluate the blood glucose control status in women with diabetes who underwent mesh removal for complications, we found that despite well-managed glucose before mesh implantation, these patients showed deterioration in glucose control over time, roughly a quarter having poor control by the time of mesh removal, and about half having poor control thereafter [45]. As it is increasingly recognized that host immune response to implants and tissue remodeling are dynamic and long-lasting, it is reasonable to recommend continuous blood glucose monitoring and management following implantation surgery to minimize hyperglycemia-associated risks in mesh complications.

Yet, glucose management is only one piece of the puzzle. We also need better tools to assess pelvic floor tissue quality preoperatively. Our animal study data, not yet published, suggest that long-duration of hyperglycemia induces thinner collagen architecture and muscularis in the vagina, along with abnormal vasculature with thinner vascular walls. These changes are further compounded by estrogen deprivation. Translating this to the clinic, long-time poorly controlled diabetes plus menopause may tell us that the patient has more fragile tissue structure, which is prone to disease recurrence, wound healing issues, and aberrant mesh–tissue integration. This scenario is worsened by hyperglycemia-skewed immune responses, leading to long-lasting inflammation and nudging the development of mesh complications. If this biology holds in humans, then the question is no longer whether diabetes matters, but how we will respond to its impact.

It is worth mentioning that adjunctive therapies—platelet-rich plasma, macrophage-based interventions, and others—have shown encouraging effects on wound healing and early inflammation resolution, though evidence specific to mesh implants remains limited [46, 47]. These approaches are not yet ready for routine practice, but they point to a paradigm in which we do more than choose “the right mesh” and “the right HbA1c.” Instead, we actively sculpt the peri-implant microenvironment to set our patients up for success.

The path forward is clear in principle: we must move beyond the binary question of “mesh or no mesh” in women with diabetes. We need a deeper understanding of how diabetes and hyperglycemic memory impact the biology of host-mesh interaction. We need mesh designs specifically fit with diabetic conditions, and adjunctive therapies that can recalibrate the immune response at the moment of implantation and beyond. We need individualized perioperative glucose management models, powered by evolving innovations in continuous glucose monitoring, biomarker discovery, genetic insights, and AI-driven modeling. Most of all, we need to bring these strands together into pragmatic, patient-centered pathways that allow women with diabetes to benefit from advanced medical devices without facing disproportionate risks.

This editorial review calls for renewed momentum in urogynecologic research, a vital step toward achieving individualized precision medicine and improving health care for women. The science is lighting the path forward; now is the time for us to follow it with purpose.
